# Implementation of the Hand hygiene self-assessment framework in a primary healthcare Centre in Saudi Arabia: A follow-up study

**DOI:** 10.1016/j.infpip.2024.100428

**Published:** 2024-11-29

**Authors:** Olaa M. Alharbi, Mohammed A. Imam, Ahmad M. Alharbi

**Affiliations:** a Department of continuing professional development and continuing medical education, Umm Al-Qura University Medical clinic, Makkah 24381, Saudi Arabia; b Department of Medical Microbiology and Parasitology, Qunfudah Faculty of Medicine, Umm Al-Qura University, Al-Qunfudah, 21961, Saudi Arabia; c Department of Clinical Laboratories Sciences, College of Applied Medical Sciences, Taif University, Taif 21944, Saudi Arabia

**Keywords:** Healthcare-associated infections, HH, HH self-assessment framework, Four moments of HH

## Abstract

Although HH (HH) practices can prevent healthcare related infections, low compliance is a major concern. We evaluated HH using a WHO observational tool and HH self-assessment framework (HHSAF) in 30 individuals in a mix of healthcare professions, before and after the implementation of the framework. In 182 opportunities to demonstrate HH practices, pre-implementation scores were assessed across five different domains including system change, training, and evaluation and feedback. Post-implementation scores obtained after 12 months showed HH compliance of 53%, with highest improvements seen across evaluation and feedback domain. The compliance rates after exposure to body fluids was 100%.

## Introduction

Healthcare-associated infections (HCAIs) are infections acquired while, or within the first 30 days after receiving health care [1]. Every year, 1.7 million hospitalised patients acquire HCAIs and one in every 17 patients dies [1]. An estimated 5%–15% of hospitalised patients acquire HCAIs in high-income countries, of which 37% acquire it when admitted to intensive care units [1]. HCAIs lead to severe infections, increased healthcare costs, and antimicrobial resistance. In South Arabia hospitals, during the 2007–2016 period a total of 1544 pathogens were involved in causing 1531 HCAIs [2]. A temporal pattern of antibiotic resistance by bacteria identified in infection causation was reported [2].

Healthcare workers are at high risk of contaminating their hands and transmitting infections. However, optimal hand hygiene (HH) behaviours effectively prevent the transmission of pathogens between patients. The World Health Organisation (WHO) has developed a HH self-assessment framework (HHSAF) to provide a situation analysis of HH resources, promotion, and practices within healthcare facilities. The usability and reliability of this tool have been confirmed to be of high standards when tested in multiple settings [3]. Implementation of the interventional HH education programme in the Emergency Department of a hospital in Saudi Arabia significantly improved compliance with HH guidelines from 30.7% to 45.5% [4]. Unfortunately, reported HH practices in Saudi Arabia hospitals are poor [5].

The national HH steering group in Saudi Arabia published the national HH standard operating procedure (SOP) in 2011. This SOP outlines the methodology for undertaking HH observational audits, which was adopted by WHO. In the present study, we aimed to implement the WHO HH standard framework to evaluate HH behaviours among healthcare practitioners in the primary healthcare Centre (PHC). The HHSAF was implemented from 01 January to 31 December 2021 and HH compliance was measured from 1 April to 30 June 2021.

## Methods

The study was conducted at Umm Al-Qura University Medical Centre, Saudi Arabia, between 01 January to 31 December 2021. This is a primary healthcare center, which consists of outpatient clinics with different sub-specialties. The centre provides consultation services, medical procedures and treatment options but without the facilities for overnight stays. Healthcare workers from different sub-specialities provide healthcare services to patients. The study was designed as a before-and-after intervention study. Pre-intervention scores were obtained before the two-day training (detailed below) to understand the existing scenario/baseline. Then the HH program was implemented. The HH compliance was finally measured as post-intervention score.

Data was collected using the Multimodal HH Improvement Strategy and the “HH Self-Assessment Framework” document (World Health Organization, 2010). The document consists of questionnaires to evaluate the following parameters: system changes, education and staff training, evaluation and feedback, reminders in the workplace, and promotion of an institutional safety climate. The scores obtained from the HHSAF are used to categorize the status as follows: Inadequate (0–125), basic (126–250), intermediate (251–375) and advanced (376–500). The healthcare units that achieve the advanced level are invited to complete an additional section (i.e., the leadership section) [6]. In evaluation and feedback, there is one indicator about direct monitoring of HH compliance using an observational tool, which was completed between 1 April 2021 to 30 June 2021 and reported as the pre-intervention score.

The compliance was assessed across four healthcare professionals - physicians, nurses, radiology technicians, and physiotherapists. The eligibility of participants included full-time staff working at the hospital for at least one year before the study commenced and they had to be previously trained for infection control.

The intervention program was then implemented. The intervention was a two-day training course consisting of two parts. The first part comprised of theoretical knowledge transfer. In this part, WHO's training PowerPoint slides for observers were shown, covering the WHO Multimodal HH Improvement Strategy and the direct observation of HH practices. The second part was a practical exercise to enable trainees to develop skills on how to conduct both HH self-assessment of the centre, and a HH audit by using a direct observation method. The WHO's direct observation form is based on “My Five Moments for HH” that consists of the following: before patient contact, before aseptic procedure, after body fluid exposure risk, after patient contact, and after contact with patient surroundings as HH indications. Appropriate or inappropriate HH action, whether hand washing or hand rubbing, was recorded if it was related to an indication. Opportunity is defined as the point in time when HH should happen, necessarily relating to at least one HH indication. The compliance is calculated by dividing positive actions by opportunities. Thirty healthcare workers participated in the study in 182 opportunities to show HH practices. A final report was provided to the centre management and heads of departments.

Both the head of the infection control department and the HH program coordinator completed the HHSAF questionnaires. We used it as the pre-intervention score. The pre-intervention score obtained was deemed low. For this reason, we implemented an observational tool afterwards between 1 April 2021 to 30 June 2021 to further assess compliance. The collected responses were checked for accuracy and completeness, and the indicators were evaluated for required improvements in the system changes, education and staff training, evaluation and feedback, putting reminders in the workplace, and promoting the institutional safety climate according to WHO framework. In December 2021, the medical director and the head of public health departments were invited to a meeting to complete the assessment and obtain the final score for the HH level by HHSAF. Two external assessors were selected to monitor the evaluation process to avoid bias. The institutional review board approved the study (Approval number: HAPO-02-k-012-2023-06-1679), and all the participants provided verbal approvals for data collection.

## Result

Thirty healthcare workers completed the study with 182 opportunities for the program monitors to observe HH behaviours. The overall HH compliance was 53%.

**Figure 1:**
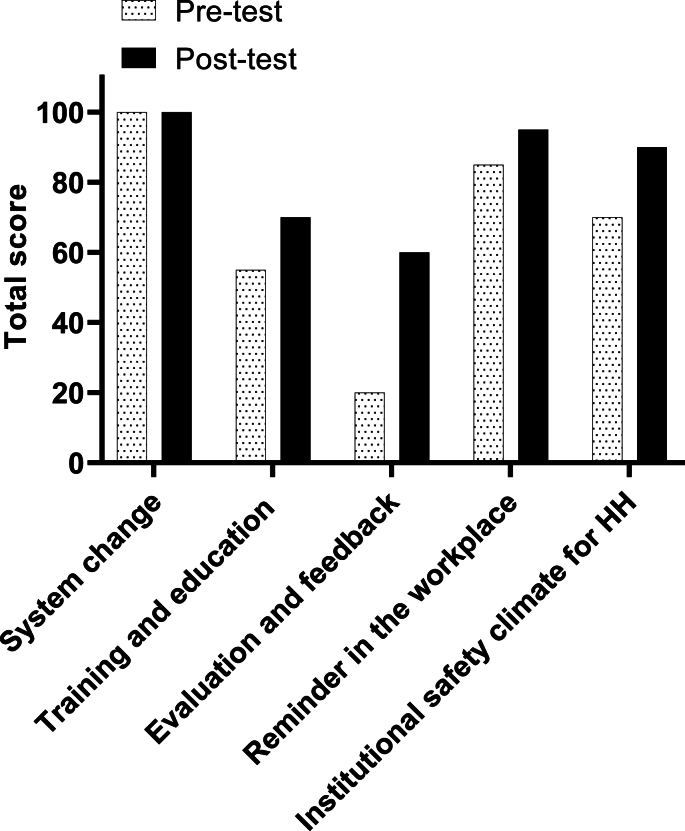
Pre- and post-evaluation scores of HH framework across the five domains of the HH module.

Scores across the five study domains are shown in Figure 1. Results show that the greatest improvement was across evaluation and feedback, in which the score changed from 20 to 60. The total score improved from 330 to 415.

Most improvements were seen across evaluation and feedback. The total score improved from 330 to 415.

### Pre and post evaluation

Pre-and post-evaluation scores matched at 100 (highest score possible) for the first domain, system change. For the second domain, there were opportunities to improve training and education for healthcare professionals. The score in this domain improved from 55 to 70. The biggest improvement was achieved across evaluation and feedback; the score improved from 20 to 60. The Primary HealthcareCentre (PHC) had reminders in the workplace, which increased further during the study period from 85 to 95. Finally, the institutional safety climate score increased from 70 to 85. It was found that the PHC needed to establish a programme of patient engagement and a system for intra-institutional sharing of reliable and tested local innovations.

### Evaluations across the Four Moments of HH

HH compliance with the ‘Four Moments for HH’ is reported in Table 1. The compliance was 31% in moment 1 (before touching a patient) and 36% in moment 2 (before a clean and aseptic procedure). The highest reported compliance was in moment 3 (after body fluid exposure risk) and moment 4 (after touching a patient), 100% and 62% respectively.

**Table 1:** HH compliance with the ‘Four Moments for HH.'

Types of indications	Times HH was performed (n)	Times observed HH indications (N)	% compliance (n/N)
Before touching a patient	25	80	31%
Before an aseptic procedure	4	11	36%
After body fluid exposure risk	1	1	100%
After touching a patient	65	105	62%
After touching patient's surroundings	0	0	0%

Abbreviation: HH; hand hygiene.

Before touching a patient, HH compliance was 52% (14 of 27 opportunities), 7% (2 of 33), 30% (3 of 10) and 60% (6 of 10) among physicians, nurses, radiology technicians and physiotherapists, respectively. Before aseptic procedures, 1/1 physician (100%), 0/6 nurses (0%), 0/0 radiology technician (0%) and 3/6 physiotherapists (50%) completed the HH procedures. On an occasion of fluid body exposure, the involved physician completed HH procedures after the exposure. None of the other professionals were involved in fluid body exposure. After touching a patient, 71% of physicians, 51% of nurses, 56% of radiology technicians and 83% of physiotherapists complied with HH practices. Overall, total HH compliance among physicians was 56% (30 of 54 opportunities), nurses were 53% (40 of 75), radiology technicians were 43% (11 of 26), and physiotherapists were 57% (16 of 28). Alcohol hand Rub was used in 81% of hand hygiene actions compared to soap and water, which was used in only 19% of hand hygiene actions.

## Discussion

The present study was conducted to understand what improvements in the organization and support provided can be improved to enhance hand hygiene. We observed that implementing the multimodal strategy at PHC in Saudi Arabia enhanced HH practices among healthcare professionals, with scores improving from the intermediate range to the advanced range. However, HH compliance was 53%. In a similar study conducted by Fouda *et al.* in Saudi Arabia, after the implementation of the interventional hand hygiene educational program, a significant improvement in compliance with hand hygiene guidelines was reported to change from 30.7% to 45.5%, which aligns with the present observation [4].

We found that HH in the PHC has reached an advanced level, although the score remains much lower than those of International standards [7]. For example, in the United States, most practices have advanced HH implementations [7]. Globally, the HH compliance rate is only 9% [8]. Our facility is in a better position than most countries but has not reached the targeted national standards. WHO reports that levels of HH compliance for even high-income countries rarely exceed 70%, requiring additional improvements [8].

In Italy, 19% of facilities were reported to achieve an advanced level, 70.4% intermediate level and 11% basic level [6]. Although most of the published literature on using the HHSAF framework encompasses big data surveillance from multiple care facilities, it is important to identify the local trends that help improve existing programs. Therefore, the present study was designed to evaluate existing HH compliance rates at PHC. Since only one healthcare facility was studied for its HH practices, the number of opportunities to evaluate hygiene practices was limited compared to multi-center studies. Nonetheless, the findings are interesting and indicate the relevance of HHSAF in our center.

The present study showed that, overall, physicians, nurses and physiotherapists complied with HH standards better with compliance rates observed between 50%-57%. Our findings are similar to data reported by Mohaithef AL *et al.* who reported that the HH standard practices were followed by 65.4% of nurses of 300 assessed in the kingdom of Saudi Arabia [5]. Generally, most staff followed hygiene protocols after touching the patient but did not do it before touching the patient. The most alarming observation was the lack of compliance among nurses, who are at the forefront of taking care of patients in a hospital. Less than 10% of nurses washed their hands before touching a patient, and only half washed their hands after touching a patient. Previously, it has been shown that with increasing work experience, nurses tend to avoid HH practices [9]. It is established that healthcare staff pay attention to HH when there appears to be a direct threat to their well-being.

Whitby *et al.* used statistical modelling to elucidate behavioural patterns of handwashing among community members and hospital nurses [10]. They described two distinct behavioural patterns, inherent handwashing (64%) and elective handwashing (76%). Nurses' handwashing practice was predicted by their beliefs in the benefits of the activity, the peer pressure of senior physicians and administrators, role modelling, and reduction in effort. They concluded that a small increase in handwashing was due to alcoholic hand rubs, which decreased the effort required. The authors argue that the introduction of hand rub alone, without an associated behavioural modification program, is unlikely to increase HH compliance. In our centre, 81% of staff used alcohol-based HH methods, which is explained by the behavioural patterns reported earlier. Also, the method reduces bacterial counts and does not affect the skin compared to plain or antiseptic soap and water. Common mistakes in the use of alcohol-based hand rubs are application to pre-irritated skin, which may cause a burning sensation. For sustainable HH practices, education targeted to modify behaviours is essential.

The present study observed that the advantages of alcohol hand rub need to be promoted at PHCs. Even with alcohol-based hand rub, a poor technique has been linked to transmitting multi-drug-resistant organisms in hospitals. Therefore, self-directed automated learning tools have been tried before to improve handwashing techniques with successful outcomes. HH training needs to be mandated at least annually, with regular monitoring of the outcome as peer pressure and administrative efforts are observed to play a role in increasing compliance. Therefore, regular auditing of the implemented HH training and programs ensures that compliance is maintained.

To conclude, the implementation of WHO's HHSAF helped to improve self-assessed components such as training and education, evaluation and feedback, reminder in the workplace, and institutional safety climate for HH. The overall HH compliance of 53% was below the national standards. Continued efforts from the administration, program evaluators, and mandatory training and audits are recommended for this center's sustainable HH level.

## Ethics approval

The present study was approved by institutional review board (Approval number: HAPO-02-k-012-2023-06-1679), and all the participants provided verbal approvals for data collection.

## Funding statement

None.

## Conflict of interest

None.
